# Mental health screening amongst police officers: factors associated with under-reporting of symptoms

**DOI:** 10.1186/s12888-021-03125-1

**Published:** 2021-03-08

**Authors:** Ruth E. Marshall, Josie Milligan-Saville, Katherine Petrie, Richard A. Bryant, Philip B. Mitchell, Samuel B. Harvey

**Affiliations:** 1 School of Psychiatry, University of New South Wales, Sydney, 2052 Australia; 2 Black Dog Institute, University of New South Wales, Hospital Rd, Randwick, 2031 Australia; 3 School of Psychology, University of New South Wales, Sydney, 2052 Australia

**Keywords:** Mental health, Psychiatry, Psychology, Post-traumatic stress disorder, Health screening, Screening, Reporting, Monitoring, Self-report

## Abstract

**Background:**

Mental health screening in the workplace aims to identify employees who are becoming symptomatic, in order to provide timely support and evidence-based interventions to those affected. Given the stigma associated with mental illness, accurate disclosure of mental health symptoms cannot be assumed. The present study sought to investigate factors associated with the accurate reporting of mental health symptoms amongst police officers.

**Methods:**

A total of 90 serving police officers completed identical mental health screening surveys, one administered by the employer and the other anonymously by an independent organisation. Responses were then linked to compare differences in the number and severity of mental health symptoms reported on each questionnaire.

**Results:**

Comparisons of matched self-report scores indicated that employees under-reported symptoms of mental health disorders when completing screening administered by their employer, with only 76.3% of symptoms declared. Under-reporting occurred regardless of gender and symptom type. Less senior staff (*p* = 0.05) and those with the most severe post-traumatic stress disorder and common mental disorder symptoms (*p* = 0.008) were significantly more likely to under-report symptoms.

**Conclusions:**

Employer-administered mental health screening is not able to accurately capture all mental health symptoms amongst first responders. The fact that the severity of symptoms predicted the level of under-reporting means that simple changes to cut-off values cannot correct this problem.

## Background

Mental health screening is often promoted as a means of helping to maintain a psychologically healthy workplace [1]. Multiple large-scale surveys have shown that nearly half of those with mental health conditions do not seek professional help, or do so only after their symptoms have begun to impact their wellbeing and workplace performance [2, 3]. Screening is therefore undertaken by many employers in an attempt to detect early symptoms of mental disorder and thus provide ready access to treatment and supports.

While the issue of delayed or otherwise deficient help-seeking for mental disorders is not contentious, the possible role of workplace mental health screening in addressing the problem has been actively debated over many years [4, 5]. The debate is particularly important for those working in roles such as first responders (police officers, firefighters and paramedics), who experience significant job-related stressors including repeated exposure to potentially traumatic events [6] [7]. Research has consistently found that first responders are more likely than the general population to experience psychological distress as a result of their work [8–10], and that they are less likely to seek professional help [11, 12]. Given their cumulative exposure to trauma alongside the prevalence of PTSD and other disorders, first responders are seen as an ideal group to receive regular mental health screening. To date, however, workplace mental health screening has not been found to be effective [13]. Similarly, the lack of evidence regarding a positive impact of screening in other high-risk trauma-exposed groups, such as the military, has raised questions about the effectiveness of mental health screening where the risk is high, but rates of help-seeking are low [14].

Recently, studies of mental health screening of police applicants by their prospective employer have shown that the measurement of established risk factors, such as early symptoms of anxiety or post-traumatic stress disorder (PTSD), did not predict future mental health problems as indicated by sickness absence for psychological injury [15]. Another study found no association between MMPI-2 scale scores, either in isolation or combination, and later psychological injury [15]. These findings have raised further doubt about the utility of mental health screening in the workplace, especially when the employer is asking the questions. In particular, the findings highlight the issue of whether or not individuals hoping to be selected as police officers are likely to answer questions about their mental health honestly. One of the reasons mental health screening fails to predict psychological vulnerability in police applicants may be the reluctance of employees or potential employees to answer questions honestly. We hypothesised that the accuracy of workplace mental health sceening would depend on how the screening is administered, specifically who is asking the questions and the level of confidentiality involved.

Public stigma attached to mental illness continues to exist in society as a whole, and particularly within male-dominated workplaces [16]. Law enforcement agencies are characterised by a male-dominated culture where a mentally tough, dependable and controlled demeanour is valued, and the expression of emotion is seen as a weakness [17]. Critically, both public stigma and self-stigma, when individuals internalise the public stigma around mental health, can act as barriers to help-seeking [18], and may prevent or delay opportunities for early intervention for officers in distress [19]. Aside from the stigma associated with mental illness, there is a broader issue of whether employees are likely to disclose symptoms of any kind to their employer, given the potential discrimination they may experience as a result [14]. Some evidence already exists that police officers are unwilling to disclose mental health symptoms to their employer fearing negative career consequences [20].

Given these issues, a key question to be answered before mental health screening programs for first responders can be considered is the likely level of honest reporting that can be expected from different groups of workers. Specifically, what are the patterns of symptom reporting and do they differ based on who is asking about mental health symptoms? Research undertaken with military personnel indicates that individuals report significantly more symptoms of PTSD, and are significantly more likely to express an interest in receiving professional support, when they are surveyed anonymously [21–23]. Fear and her colleagues (2012) found the stigmatising beliefs that “leaders discourage the use of mental health services”, “it would be too embarrassing”, and “I would be seen as weak” were significantly more likely to be endorsed anonymously; and highlight that such beliefs are clear barriers to disclosure and subsequent help-seeking [22]. Similar studies have not been carried out amongst first responders. It is also unclear if this pattern is seen amongst all workers, or whether different groups are more or less likely to under-report symptoms. It might be hypothesized, for example, that staff at a higher rank would be more prepared to disclose mental health symptoms given their greater degree of job security, or that female staff would be more likely to seek help in line with gender norms. The type of occupation and workplace culture may also affect employee’s willingness to disclose mental health symptoms, particularly in occupations such as first responders, though this has not been examined to date. Such information is needed to inform the design of more effective mental health screening programs.

The aim of this study was to compare responses provided by serving police officers to identical mental health questionnaires asked in two different ways; by their employer and confidentially via a research team. The study then aimed to investigate factors which may impact on the level of disclosure, such as gender, age, rank, type or severity of symptoms.

## Method

### Participants

Participants for this study were recruited from an Australian police force. Participants were drawn from trial sites of a new voluntary online mental health screening program being implemented by that organisation. All procedures were approved by the Human Research Ethics Committee at the University of New South Wales (HC14197). Informed consent was obtained from all participants via an online form approved by the UNSW Human Research Ethics Committee.

### Research design

The study involved linked cross-sectional surveys of mental health symptoms measured by identical validated questionnaires, one employer-administered as part of the organisation’s screening program and one administered within 6 weeks by independent researchers.

### Procedure

Serving police officers were invited via e-mail by police psychologists to participate in the organisation’s mental health screening program. This program was administered by the police force was not anonymous, and participants were aware that individuals reporting significant levels of symptoms may be offered a follow-up assessment and counselling by a police psychologist, replicating the process undertaken as part of most workplace mental health screening programs.

On completion of the organisation’s mental health screening program, all eligible police officers were informed that independent researchers from the University of New South Wales (UNSW) would be conducting a separate confidential survey to evaluate the new program. Police officers were assured that, as this was an independent project, their professional anonymity would be maintained. Within 6 weeks of completing the employer-administered mental health survey, respondents were e-mailed by the UNSW research team. This e-mail invited them to participate in an online validation questionnaire via QuestionPro and provided a consent form and unique URL link to access the survey. Police officers were again reassured regarding the confidentiality of their participation and results, specifically stating that any information provided would not be made available to their employer in an identifiable way. Responses from the police screening program were linked with responses from the UNSW validation questionnaire using the unique survey URLs and police officers’ staff identification numbers.

The police screening program and subsequent UNSW validation questionnaire both contained two identical mental health measures: the short-form Depression Anxiety Stress Scales (DASS-21) [24] for symptoms of psychological distress, and an abbreviated version of the PTSD Checklist for DSM-5 (PCL-5) [25] for symptoms of post-traumatic stress disorder (PTSD). The DASS-21 consists of 21 items relating to low positive affect, physical arousal, and tension/irritability and is scored from 0 (never) to 3 (almost always) [26]. The DASS-21 is a valid measure of depression, anxiety, and stress symptoms, and is suitable as a routine clinical outcome measure for psychiatric patients [27] and as a screening tool for possible common mental disorder (CMD) [24]. Internal validity of the DASS-21 was high (Cronbach’s alpha = 0.963). The abbreviated version of the PCL-5 consists of four items corresponding to each of the symptom clusters represented in the DSM-5 PTSD diagnosis, scored from 0 (not at all) to 4 (extremely) [28]. The 4-item PCL-5 is a valid measure of PTSD symptoms and is highly correlated with the full PCL-5 in community samples of trauma-exposed adults [28]. Internal validity of the PCL-5 was high (Cronbach’s alpha = 91.2%).

### Data analysis

Data were analysed using STATA 12.1. Paired samples t-tests were conducted to investigate differences in the level of mental health symptoms reported on the employer-administered screening compared to the confidential questionnaire. Independent samples t-tests were conducted to investigate any differences in percentage of symptoms overall, as well as looking at under-reporting due to gender (F/M) or age (18–44 years/45–70 years). Symptom severity was determined by summing the symptom totals and dividing into tertiles subsequently referred to as Low, Moderate and High. Rank and symptom severity were then analysed using univariate linear regression to investigate any association with the under-reporting of mental health symptoms.

## Results

Of the 694 police officers invited, a total of 412 police officers completed at least one of the surveys (study response rate 59%). A total of 261 police officers completed the e-Well Check employer-administered screening only, 53 completed the anonymous independently-administered survey only, and a total of 90 serving police officers completed all questions on both mental health screening surveys. Table 1 provides a description of the sample. As highlighted by the age and rank of participants, our sample had a mixture of new and experienced police officers. The mean age of those who completed both surveys was 44.1 years (SD = 9.3), with 68.89% (62/90) male. These demographics, most notably an over-representation of males, are similar to those for the entire organisation.

**Table 1:** Description of the sample of police officers used in this study

Variable	Number	% of sample
Gender		
Female	28	31.11
Male	62	68.89
Age		
18–24	2	2.22
25–34	12	13.33
35–44	25	27.78
45–54	45	50.00
55–70	6	6.67
Rank		
Constable	1	1.11
Senior Constable	22	24.44
Sergeant	23	25.56
Senior Sergeant	6	6.67
Inspector	17	18.89
Superintendent	3	3.33
No data	18	20.00

**Fig. 1:**
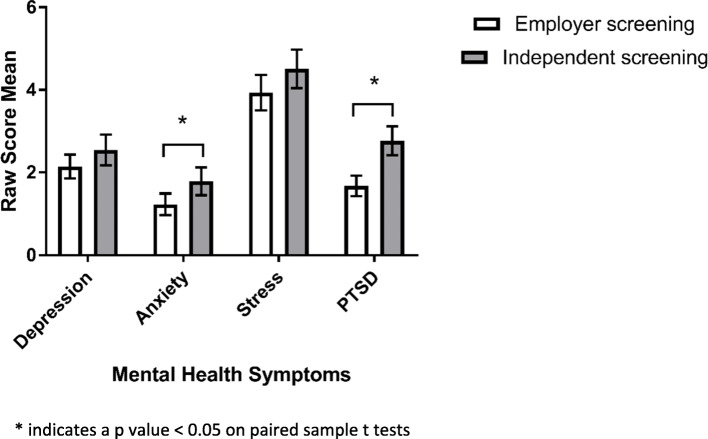
Level of mental health symptoms reported by the same individuals completing both employer-administered and independent mental health screening

As shown in Fig. 1, police officers reported significantly lower levels of anxiety (*p* < 0.05) and PTSD (*p* < 0.001) symptoms on the employer-administered survey compared to the confidential survey. There were no significant differences in the levels of depression or stress symptoms self-reported via the two methods although similar trends were apparent. Overall, police officers only reported 76.3% of the mental health symptoms that they declared on the independent screening within the employer-administered questionnaires.

**Fig. 2:**
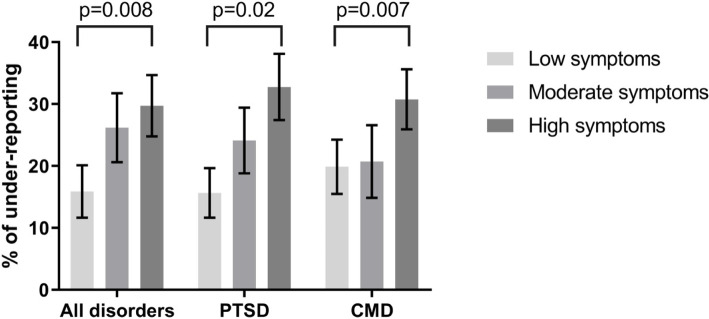
Rate of mental health symptom under-reporting on employer screening (compared to independent screening) based on levels of symptom severity

As demonstrated in Table 2, gender did not predict the level of symptom reporting to employers, with both male and female officers under-reporting symptoms at a similar level (*p* = 0.83). Rank rather than age made a difference to the amount of under-reporting, with those at higher ranks reporting more of their symptoms on the employer-led survey (*p* = 0.05). As shown in Fig. 2, results indicate that police officers with the most severe symptoms were significantly more likely to under-report symptom to their employer (*p* = 0.008). This was the case for both PTSD symptoms (*p* = 0.02) and CMD symptoms (*p* = 0.007).

**Table 2:** Factors associated with accurate symptom reporting on workplace mental health screening

Variable	Number	% of symptoms reported (95% CI)	Significance ***p*** value^1^
Gender			
Female	28	77.2 (66.8–87.6)	0.83
Male	62	75.8 (68.9–82.9)	
Age			
18–44	39	74.8 (65.6–84.0)	0.65
45–70	51	77.4 (70.0–84.9)	
Rank			
Constable/Senior Constable	23	64.9 (50.8–79.0)	0.05
Sergeant/Senior Sergeant	29	79.3 (69.8–88.8)	
Inspector/Superintendent	20	81.6 (70.3–92.9)	
Symptom severity^a^			
Low	32	84.1 (75.5–92.7)	0.008
Moderate	28	73.8 (62.4–85.2)	
High	30	70.3 (60.1–80.4)	

^1^ *p* values obtained from t-test for gender and age and from univariate linear regression for rank and symptom severity

^a^ Summed symptom totals divided into tertiles

## Discussion

Workplace mental health screening to both detect symptomology and intervene early is widely practiced amongst first responder agencies with limited evidence to support its effectiveness [29]. One reason for the ineffectiveness of workplace mental health screening may be that respondents under-report symptoms when they are aware that their responses are being collected by their employer. The aim of the current study was to test this hypothesis by comparing the number and severity of mental health symptoms reported by serving police officers in an employer-administered questionnaire, and in the same questionnaire when administered anonymously by an independent organisation. Comparisons of matched self-report scores indicated three patterns. Firstly, there was a general trend for employees to under-report symptoms of mental health disorders when screening was administered by their employer, with only three-quarters of symptoms being disclosed. This pattern was present regardless of gender. Secondly, less senior officers appear to have found it particularly difficult to report their symptoms, with rates of reporting falling to under 65% for constables and senior constables. Finally, and perhaps most importantly, the level of under-reporting was closely associated with the overall number of symptoms. Those with the most severe PTSD and CMD symptoms were significantly more likely to under-report, meaning that those most in need of help, were least likely to be identified in employer-led screening.

Despite calls for further research to establish the effectiveness of mental health screening amongst first responders in recent years [29, 30], this is the first time that identical employer-administered and independently administered screening tools have been directly compared within an emergency service organisation.

There are some limitations that need to be considered prior to interpreting these results. First, although we were able to demonstrate statistically significant differences within the data analysis, it is possible that the relatively small sample size limited the statistical power necessary to detect other important relationships. The 18 participants who didn’t want to disclose their rank were excluded from the univariate linear regression analysis. This would have reduced statistical power and increased the risk of type II error, but despite this our results were significant.

Secondly, our sample consisted only of police officers, and our results may not be generalised to other emergency personnel. Indeed, studies have found that the majority of police officers, particularly early in their career, are psychologically well-adjusted [31] and have better mental health that the general population [32]. Generalisability of the results could also be questioned as those responding to both questionnaires opted in knowing that this was a mental health survey, arguably because they felt more comfortable talking about mental health concerns. If that were the case, however, our results would likely be an underestimate of the true problem, as those opting out of completing mental health surveys may be more uncomfortable around disclosure and even more likely to under-report symptoms in employee-led questionnaires.

Thirdly, when comparing responses given on identical mental health questionnaires, it is difficult to ascertain whether it was the agency who administered the screening, or the level of anonymity offered to respondents that accounted for the differences observed. However, while employees may have been happier to disclose their symptoms to an employer offering the same level of anonymity as an external agency, they may be more suspicious of a workplace claiming total anonymity.

Fourthly, this study used brief validated measures of PTSD and psychological distress symptoms, but we were not able to explore differences between the individual symptoms endorsed by respondents in a subsequent clinical interview. It may be that some items, such as flashbacks, are more readily disclosed than others, such as low mood. Further, although we were able to assess the severity of symptoms, we were unable to ascertain how long an individual had been experiencing them, or whether or not they had sought help.

Fifth, the questionnaires were completed up to 6 weeks apart, so the differences in reported mental health symptoms may represent fluctuations over time or exposure to additional traumatic incidents, rather than a systematic difference. This is unlikely, however, as changes over time would include increases as well as decreases in symptom scores. Replication with larger samples of police officers and other high-risk groups with repeated assessments conducted over time could inform such issues. Finally, there was a fixed order in which employer administration and confidential administration of the symptoms occurred. This is potentially problematic because completing the assessment in response to the employer-administered survey may have primed police officers’ awareness of their mental health, which may have contributed to their subsequent elevated reporting of symptoms during the confidential survey. To address this, future replications of this study should administer employer and independent formats of assessment in a counter-balanced order to eliminate the possibility of priming effects confounding interpretation. This procedural modification would also address the issue of symptoms fluctuating over time and the possibility of exposure to potentially tramatic events in the period between the first and second surveys.

In considering our results, it remains possible that the cause of the differences observed might not be that people under-report symptoms on a questionnaire that will be seen by their employer, but that they over-report symptoms on an anonymous questionnaire [22]. This would appear unlikely as there would be no benefit to exaggerating symptoms anonymously, but every reason to under-report symptoms in a workplace-administered test for fear of negative career consequences. While motives to disclose or withhold information could only be confirmed by interviewing respondents, Fear et al. point to previous studies also showing a tendency to under-report PTSD symptoms amongst serving military personnel [21, 23]. These studies similarly surveyed existing employees, and one might argue that the difference observed would be even larger in a pre-employment context, where individuals are being screened for hiring purposes. As far as we are aware, no study of military or emergency services personnel has looked at this. Given our finding that that those of a higher rank were less likely to under-report symptoms, one could argue that honest disclosure might be related to increased job security.

## Conclusion

Our findings highlight the key challenge for emergency services organisations if they attempt to screen individuals for symptoms of PTSD and CMD. Mental health screening in high-risk occupations aims to maximise accurate identification of those suffering symptoms in order to enable the timely provision of evidence-based interventions. However, if employees are unwilling to disclose these symptoms to their employer then it becomes more difficult to direct resources to those in need. In addition, our key finding that the level of under-reporting is greater as the severity of symptoms increases, would mean that simple measures such as adjustment of usual cut-off points for questionnaires, will not be effective in delivering a more accurate assessment. While our findings suggest that police officers are more likely to respond honestly to a confidential questionnaire, organisations whose role is to protect the public may be reluctant to leave help-seeking to the individual without the ability to monitor their psychological well-being and ongoing capacity to undertake their work duties. Our findings are not able to answer the question of whether mental health screening is effective. This will require large scale intervention trials. However, our results do provide important insights into what factors may make mental health screening more likely to be effective in the workplace. In the end, whether to use mental health screening and how to administer this is going to depend on the underlying purpose. If screening is being carried out to ensure that first responders are able to undertake their work in a safe way, the employer-led non-anonymous screening, with all its limitations, remains the only option. If, however, the primary motivation for mental health screening is to promote early help-seeking amongst first responders, then these results suggest that independent confidential screening is likely to be more effective.

## Data Availability

The datasets generated during and/or analysed during the current study are available from the corresponding author upon reasonable request.
